# Beyond the double helix: DNA structural diversity and the PDB

**DOI:** 10.1016/j.jbc.2021.100553

**Published:** 2021-03-17

**Authors:** Stephen Neidle

**Affiliations:** The School of Pharmacy, University College London, London, UK

**Keywords:** DNA, deoxyoligonucleotides, crystal structures, NMR, sequence-dependent structure, double helix, multistrand helices, quadruplex, PDB, NDB, NDB, Nucleic Acid Database, PDB, Protein Data Bank

## Abstract

The determination of the double helical structure of DNA in 1953 remains the landmark event in the development of modern biological and biomedical science. This structure has also been the starting point for the determination of some 2000 DNA crystal structures in the subsequent 68 years. Their structural diversity has extended to the demonstration of sequence-dependent local structure in duplex DNA, to DNA bending in short and long sequences and in the DNA wound round the nucleosome, and to left-handed duplex DNAs. Beyond the double helix itself, in circumstances where DNA sequences are or can be induced to unwind from being duplex, a wide variety of topologies and forms can exist. Quadruplex structures, based on four-stranded cores of stacked G-quartets, are prevalent though not randomly distributed in the human and other genomes and can play roles in transcription, translation, and replication. Yet more complex folds can result in DNAs with extended tertiary structures and enzymatic/catalytic activity. The Protein Data Bank is the depository of all these structures, and the resource where structures can be critically examined and validated, as well as compared one with another to facilitate analysis of conformational and base morphology features. This review will briefly survey the major structural classes of DNAs and illustrate their significance, together with some examples of how the use of the Protein Data Bank by for example, data mining, has illuminated DNA structural concepts.

A well-known protein crystallographer told me, almost exactly 50 years ago, that “DNA structure is monotonous and boring.” This assertion can be taken to mean that (i) the double helix appears to be invariant along the length of the genome, so that DNA structure is fully represented by the Watson–Crick model and thus would not be of any future interest, and consequently, (ii) the future study of DNA structure is an inherently uninteresting topic. The subsequent history of the subject has comprehensively disproved both these statements—it has also turned out that DNA structural studies continue to have a flourishing existence well beyond the double helix, with distinctive and hitherto unimagined novel structural types being discovered and having major biological significance. Highlights of these varieties of structures will be discussed in this brief review. A more comprehensive and detailed account of DNA structures can be found elsewhere (1).

It is inconceivable that any structural studies on DNAs over the past 50 years would have taken place without the involvement of the Protein Data Bank (PDB) at some point. All crystal and NMR structures are deposited in the PDB and released for open access, normally either before to or immediately after publication. For any new structure, comparative studies with existing structures are an essential part of any meaningful analysis, and the PDB has long provided the data and tools for these to be undertaken. For DNA structural studies, the PDB is, though, much more than a passive depository of structures to be uploaded or downloaded as needed. This article aims to highlight some of the major steps in our knowledge of DNA structure because the advent of the double helix concept and how the PDB has, in various ways, played a key role in actively facilitated these advances. This role, and that of the associated Nucleic Acid Database (NDB), is discussed in more detail at the end of this review. Readers are encouraged to browse through some of the structures highlighted here. To this end, Table 1 details some representative DNA crystal structures and includes their unique PDB ID numbers and hyperlinks to the PDB.

**Table 1:** Selected DNA crystal structures highlighted in this review

Sequence	Structure type	PDB ID	Resolution, Å	Ref
d(CGCGAATTCGCG)	B-DNA double helix	1BNA	1.9	(15)
d(CGCGAATTCGCG)	B-DNA double helix	436D	1.1	(17)
d(CGCGAATTCGCG)	B-DNA double helix	4C64	1.32	(18)
d(CGAATTAATTCG)	B-DNA double helix	5M68	2.64	(22)
d(ACCGAATTCGGT)	Bent B-helix A-tract	1ILC	2.2	(33)
d(CGCGAATTGGCG)	B-DNA + G:G mismatches	1D80	2.2	(36)
d(CTACGCGCGTAG)	A-DNA double helix	5MVK	1.5	(38)
d(CCGGGCCCGG)	Holliday junction	1ZF2	1.95	(43)
d(CGCGCGCGCGCG)	Z-DNA double helix	4OCB	0.75	(48)
d(GGGTTAG^Br^GGTTAGGGTTAG^Br^GG)	Antiparallel chair telomeric quadruplex	6JKN	1.40	(65)
d(GGGCGGGGAGGGGGAAGGGA)	*BRAF* quadruplex	4H29	1.99	(77)
d(AGGGAGGGCGCTGGGAGGAGGG)	c-*KIT* quadruplex	4WO2	1.82	(76)
d(TGAGGGTGGGTAGGGTGGGTAA)	c-*MYC* quadruplex	6AU4	2.35	(78)
d(AGGGCGGTGTGGGAATAGGGAA)	*KRAS* quadruplex	6N65	1.6	(79)
d(TGGTGGTGGTGGTTGTGGTGGTGGTGTT)	Left-handed quadruplex	4U5M	1.5	(81)
d(ATCCGATGGATCATACGGTCGGAGGGGTTTGCCGTTTAAGTGCC)	Deoxy-ribozyme	5CKK	2.8	(91)
23 x d(TTAGGG)	Human telomeric nucleosome	6KE9	2.22	(105)

PDB, Protein Data Bank.

This review focusses on crystal rather than NMR structures, in part in view of the ability of high-resolution crystallography to visualize the essential role of water in maintaining DNA structural integrity, as well as acknowledging the central role played by crystal structures in the historic development of our understanding of DNA structure. Structural analysis of DNA-small molecule complexes is a subject in its own right and is not covered here; as with native DNAs, the PDB continues to play a critical role in the dissemination and analysis of these structures.

### Some background

The determination of the structure of the genetic material, double-helical DNA, by Watson, Crick, Franklin, and Wilkins in 1953 (2, 3, 4) is by common consent the key landmark in the development of modern biological and biomedical sciences. This was also the first macromolecular biological structure to be determined at an “atomic” level. It is worth reminding ourselves, almost 7 decades on from that momentous work, exactly what this structure determination does (and does not) tell us about DNA. It used the methodology of X-ray fiber diffraction, which relies on aligned and semicrystalline arrangements of polymeric DNA molecules to produce diffraction patterns that represent the average of all sequences in that DNA. The structure was determined, not by *ab initio* crystallographic methods, but by model-building, comparing a plausible molecular model for the structure with the observed fiber diffraction patterns. This resulted in 1953 in a structure with key features, notably of an antiparallel right-handed double-helix arrangement, “Watson–Crick” A:T and C:G base pairing (Fig. 1*A*), a 3.4 Å base pair repeat and exactly 10 base pairs per helical turn, that best fitted the (B-DNA) fiber diffraction data. Sequence-dependent structural information at the individual nucleotide and base pair level is unavailable from this approach. Inevitably, there were some (albeit a small minority) who questioned the validity of the antiparallel Watson–Crick base-paired double helix concept in view of its reliance on molecular modeling, rather than being the result of purely crystallographic analyses. A subsequent series of careful quantitative analyses and structure refinements of both A- and B-DNA fiber diffraction structures (as well as of other natural and synthetic DNA and RNA polynucleotide fibers) by Arnott, Fuller, Wilkins and their colleagues (5, 6) did much to dispel doubters. The ramifications of the double helix concept for biology and genetics have been profound and are fully consistent with the model. However, a formal crystallographic “proof” of the double helix concept that does not have any inbuilt assumptions only became available in 1980, 27 years after the first announcement of the structure. Fiber diffraction is an excellent technique for studying the polymorphism of DNA (and RNA) random- and repetitive-sequence natural and synthetic polynucleotides at moderate resolution, up to ca 2.5 Å. However, it is inherently unable to examine effects at the local nucleotide or base pair level. By contrast, single-crystal studies (together with NMR) have enabled over 2000 oligonucleotide structures to be unequivocally determined, covering a wide range of DNA sequences (and diverse structures). These are increasingly at atomic-level high resolution, occasionally even at <0.7 Å, enabling the finest points of detail to be defined, not least sequence-dependent properties, base tautomerism, base-base hydrogen bonding, and water networks associated with DNA.

**Figure 1:**
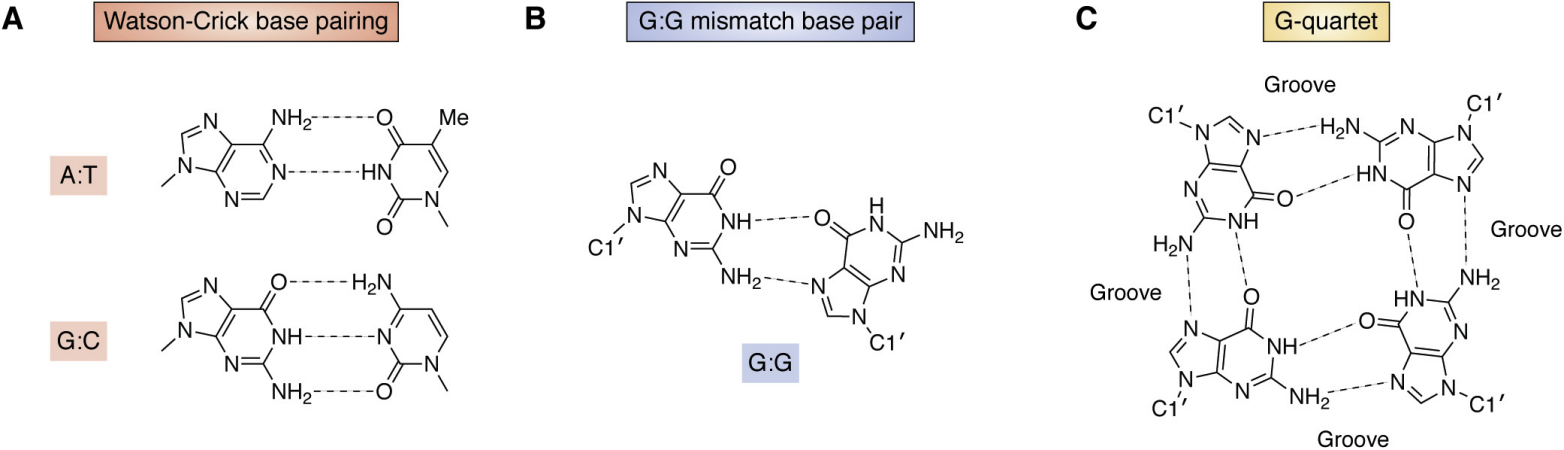
**Various forms of base–base hydrogen bonding observed in DNA crystal structures.***A,* classic Watson-Crick base pairing as found in unmodified duplex DNAs. *B,* an example of a base-pair mismatch, as found in some G.G mismatched duplex DNAs. *C,* the arrangement of eight Hoogsteen hydrogen bonds between the four guanine bases in a G-quartet.

It has been for long realized that the inherent limitations of fiber diffraction cannot provide atomic-level information and data on the effects of particular sequences on the double-helix structure of genomic DNA because as stated above, the technique averages structural information over all sequences present in a fiber. The simplest repeating unit that could possibly reveal some detail at the individual nucleotide level is a dinucleotide (or a dinucleoside monophosphate), possessing the key 3′-5′ sugar phosphate linkage. The first single-crystal determination of such a sequence was in 1971, of the ribo-dinucleoside phosphate r(UA) in 1971 (7, 8, 9), which crucially did not rely on a preconceived model for structure determination. This (RNA) fragment although not forming a conventional double helix revealed several novel features about nucleic acid conformation and paved the way for subsequent studies on other helical fragments (see below). Some (but not all) of these early dinucleoside crystal structures were deposited in the Cambridge Crystallographic Data Base—those that were not, appear to be lost to posterity.

The definitive validation of the structure of the DNA double helix did not occur until oligonucleotide synthesis at the multimilligram level became feasible and ultimately widely available. This advance enabled single-crystal studies of many defined-sequence oligonucleotides, from the 1970s onward. They have revealed a richness of detail that was and still is, unavailable from fiber diffraction studies.

## The double helix visualized by single-crystal studies

Two seminal crystal structures (10, 10, 11, 12, 13), again of self-complementary ribo-dinucleoside phosphates, the sequence r(GC) and r(AU), were the first to demonstrate the existence of Watson–Crick base pairs (Fig. 1*A*) within antiparallel double helices, albeit short two-base pair ones. These are not short DNA but RNA helical fragments whose helical appearance and parameters are satisfyingly in accord with earlier fiber diffraction analyses of natural and synthetic double-stranded A-RNA type polyribonucleotides, having base pairs inclined with respect to the helix axis. The average helical twist angle of 32.5° in these two double-helical fragment structures is compatible with a standard A-type helix, having an average of 11 base pairs per complete helical turn (14).

**Figure 2:**
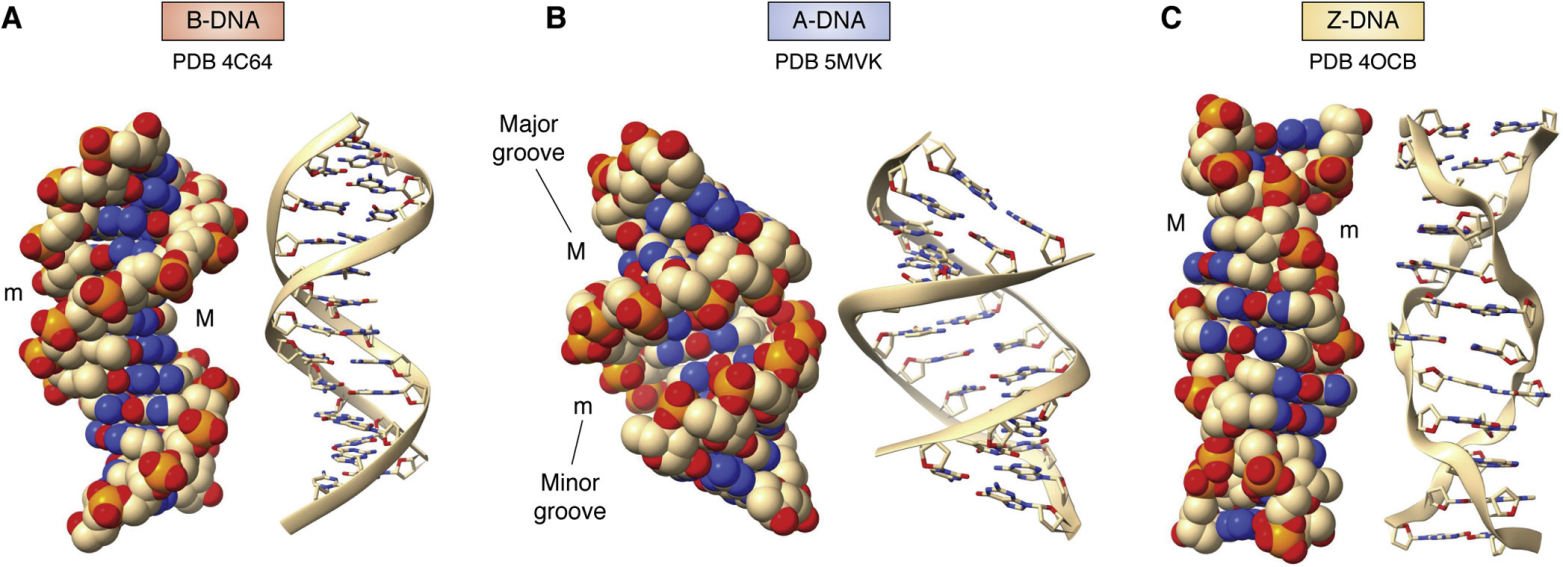
**Crystal structures of B, A, and Z form double helices, each showing space-filling and cartoon representations. Major (M) and minor (m) grooves are indicated.***A*, the structure of the B-DNA Dickerson-Drew dodecamer (18). The two representations are taken from identical viewpoints, with each showing the narrow minor groove in the *top part* of the helix and the wide major groove in the *lower part*. These and all subsequent molecular structure figures have used the ChimeraX molecular graphics program (19). *B*, representations of an A-DNA dodecamer crystal structure (38), showing an approximately 11-fold helix with the characteristic narrow major groove at the center of the view. Base pairs are tilted with respect to the (vertical) helix axis. Although, even though there are local variations in base and base pair morphological parameters, the overall arrangement is close to an A-DNA helix derived from fiber diffraction. *C*, representations of the crystal structure of the left-handed Z-DNA sequence d(CGCGCGCGCGCG) (48). Note the characteristic irregular zig-zag feature of the phosphodiester backbone shown in ribbon cartoon form. PDB, Protein Data Bank.

**Figure 3:**
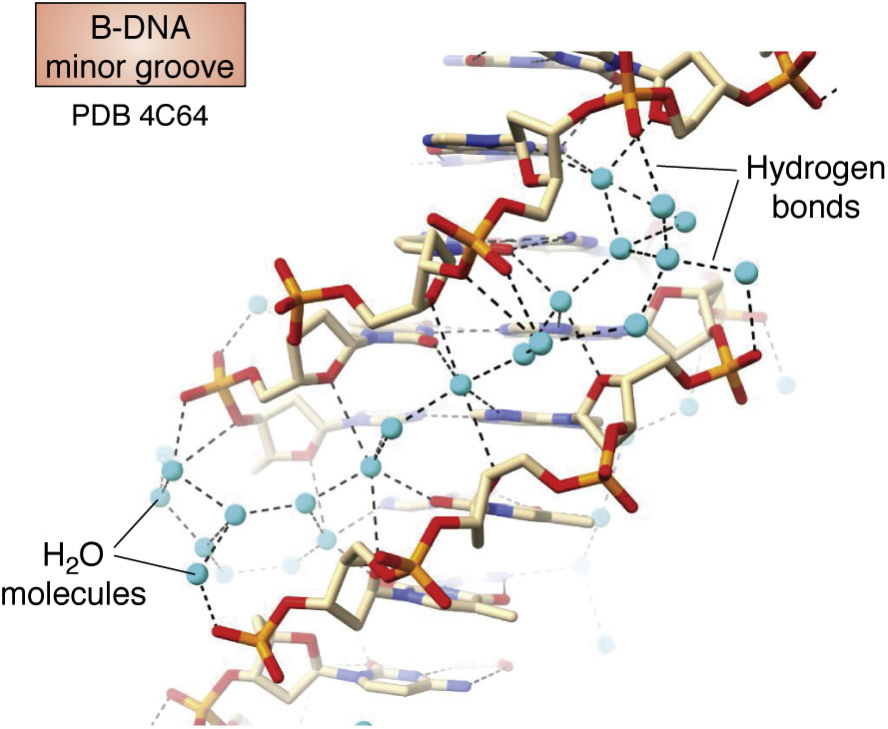
**Detail of the water structure in the B-DNA dodecamer minor groove** (18)**, showing the water molecules (in *cyan*) and hydrogen bonds to waters and DNA.** PDB, Protein Data Bank.

Wing *et al.* (15) reported in 1980 the first crystal structure of an oligonucleotide displaying a full helical turn, determined by the classic isomorphous replacement methods of protein crystallography, so that there was no bias in the structure from any preconceived structural model. This structure is of the self-complementary dodeca-deoxyribonucleotide d(CGCGAATTCGCG). Two strands associate together to form in the crystal (and in solution) a B-type DNA Watson–Crick base-paired antiparallel double helix (Fig. 2*A*), albeit with helicity slightly greater than the 10 base pairs per turn in the exactly repetitious fiber diffraction B-DNA model. These features of this, the so-called “Dickerson-Drew” dodecamer, constitute a formal atomic-level validation of the original Watson–Crick model for B-DNA. The B-type helix is still considered to be the most representative form for most of the DNA in the human genome. This and subsequent crystal structures have also revealed much more than the “monotonous” double helical features in the fiber diffraction model. Most significant are the sequence-dependent features of flexibility and variations in base pair morphology and backbone conformation, as highlighted in the central four base pair d(AATT) region of the Dickerson-Drew sequence, where there is a narrowing of the minor groove width. As a consequence, a highly structured water network is localized in this groove (Fig. 3), which is termed the “spine of hydration”, with hydrogen bonds to phosphate groups and O4’ sugar ring atoms at the mouth and walls of the groove and to base edges on the floor of the groove (16). Since the original structure determination, at 1.9 Å resolution, many further analyses of this sequence at higher atomic-level resolution, and with more modern crystallographic refinement methodology, have been undertaken (see for example: refs (17, 18)). The structure shown in Figure 2*A*, at 1.3 Å resolution, is typical of these, and the fundamental sequence-dependent features are retained in them, not least the spine of hydration.

### DNA sequence-dependent structure

An A-tract is defined as a short run of adenosine residues, often within a longer sequence, for example d(AAAA) within the sequence d(CGAAAATTTTCG). It has been suggested that the structural features of the A-tracts seen in the Dickerson-Drew and other DNA crystal structures are a consequence of crystal packing forces rather than being intrinsic properties of DNA local structure. A feature of the original native dodecamer Dickerson-Drew crystal structure is that its orthorhombic crystal packing involves the ends of one molecule interlocking with another, potentially constraining the ability of the central region to deform according to sequence and crystal packing effects. Surveys of the many such dodecamer crystal structures in the PDB (for example, in refs (20, 21)) have revealed that the effects of intermolecular helix–helix interactions are actually small compared with the other forces involved, principally intramolecular base-base stacking and base edge–edge repulsions. This issue has also been addressed by dodecamers and other length DNA duplexes being crystallized in a variety of space groups. For example, duplex packing in the trigonal space group *P*3_2_ which is sometimes observed when co-crystallized with nickel (2^+^) ions involves end-to-end pseudo-stacked helices running through the crystal structures, such as that of d(CGAATTAATTCG) (22). This arrangement releases the base pairs near to the helix termini to be free from any potential crystal packing constraints.

**Figure 4:**
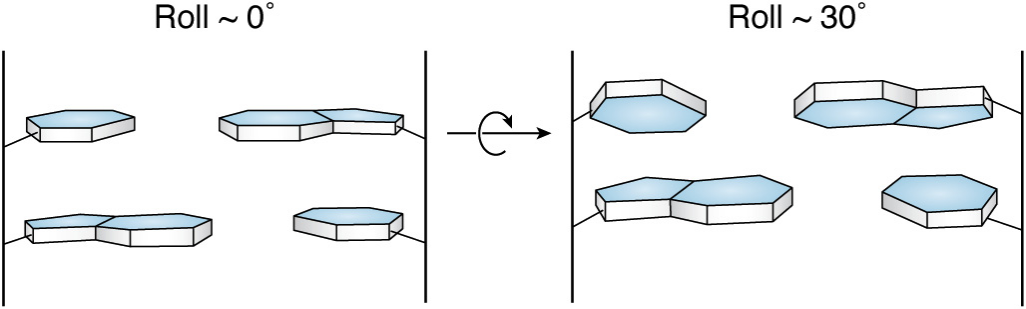
**A schematic representation of roll between two successive base pairs.** LHS: the roll is ca 0°. RHS: the roll, of ca 30°, is opening the base pair toward the minor groove, *i.e.*, toward the viewer. The cross-over point is indicated by an *arrow*.

Broader questions of the relationships between local DNA sequence, including purely AT ones, and structure have been explored in numerous subsequent structural, biophysical, and theoretical studies of other DNA sequences, often using the structures in the PDB to generate data for particular base steps and sequence variations. Sequence-dependent features require a number of morphological parameters to fully describe base and base pair flexibility and for qualitative and quantitative analyses. These parameters include base pair propeller twist, roll (Fig. 4), and helical twist between successive base pairs: a set of unambiguous definitions of these parameters, initially for duplex DNA and RNA, was agreed in 2001 at a meeting convened by the NDB (23). Computational tools are now available for calculation of these morphological parameters (24, 25), which are for the most part also available directly from the NDB. As far as biological DNA in chromosomes is concerned, the overall view of duplex DNA being uniformly smoothly B-form is thus updated because of local variations in these parameters, which are dependent on sequence and sequence context. Several sets of rules have been formulated to explain these local variations in terms of responses by individual bases, base pairs, and base steps to intramolecular clashes between neighboring atoms and groups. The early Calladine-Drew rules (26) were in large part based on the data from the original Dickerson-Drew crystal structure. Subsequent extensions of these rules have taken data from other more recent DNA crystal structures in the PDB, as well as from high-quality molecular dynamics simulations (27). Base, base pair, and base-step local structure also play a key role in understanding DNA-protein recognition and consequent function (28). It should be borne in mind that the accuracy and precision of many (not least DNA) crystal structure determinations has improved with time, in line with improvements in (i) X-ray source intensity and detection, which have led to improved data quality and higher resolution and (ii) in refinement techniques and parameterization, so enabling improved accuracy and precision in these derived parameters.

Dodecanucleotide and decanucleotide crystal structures have also been widely used as templates for numerous structure analyses probing changes in sequence in the central hexanucleotide or octanucleotide region to examine sequence-dependent properties in these central base pairs such as:

(i) DNA bending (29), which is an essential requirement for duplex DNA in many interactions with proteins, especially those involved in gene regulation (30). Early crystal structures of dodecamers with A-tracts of sequence d(A_n_T_n_) showed significant intrinsic bending toward the minor groove direction (see for example, refs (31, 32)), with the A-tracts themselves being straight. Changes in base pair roll, buckle, and/or tilt at the A-tract ends result in bending at the junctions with general-sequence DNA, as shown for example by the crystal structure determination of d(ACCGAATTCGGT). This has three independent B-form dodecamers in the crystal structure (33). Bending in all three helices is closely similar and is in accord with solution data, and detailed analysis was able to discount any influence of crystal packing forces on bending.
(ii) The effects of base mismatches. Many structures incorporating mismatches have been examined, exploring most possible mismatches (34). Examples include purine:purine mismatches such as the A:G and G:G (Fig. 1*B*) base pairs, both of which can be accommodated within duplex DNA with only minor distortions, chiefly backbone bulges and reduced stacking between mismatch and adjacent Watson–Crick base pairs (35, 36). The engineering of base pairs using synthetic bases of defined sizes that can also result in “skinny” (using base pairs constructed from two small bases) and “fat” helices (with two large bases) (37).

### Other right-handed helices

Two other helical forms of DNA are notable. Numerous A-form DNA crystal structures have been determined, for a range of oligonucleotide lengths. These tend to adopt the A- and A'-DNA features found by fiber diffraction, for example as found in the high-resolution structure of the d(CTACGCGCGTAG) duplex (38) (Fig. 2*B*) and hence have provided validation for the early A-form assignments of fiber DNA analyses (1, 39). The A-form structures have wide, shallow minor grooves, approximately 11-fold helices, base pairs inclined to the helix axis and sequence-dependent local geometry. It is not uncommon for a sequence to crystallize in an A-form, yet as shown by NMR methods, the B-form dominates in solution. Thus, the presence of the A-form in the crystal is sometimes a consequence of crystal packing forces (38). However, the A-form is not an artefact. A-DNA polynucleotide fibers are produced in lower humidity environments than B-DNA (4, 39), and the A-form as observed both in single crystals and fibers has been found in appropriate environments for example in the genomes of some double-stranded DNA viruses that have evolved to be protected from excess hydration (40).

**Figure 5:**
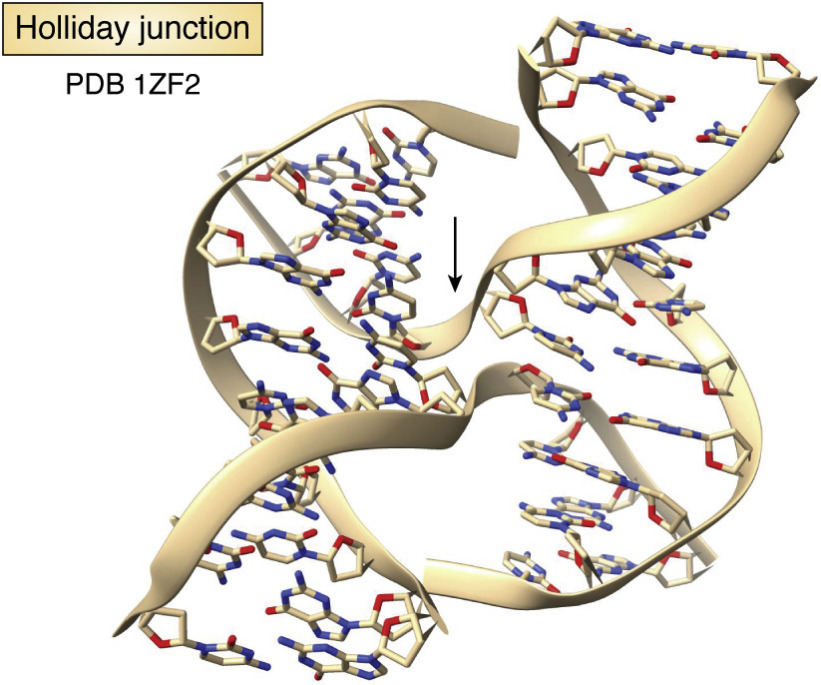
**Structure of a Holliday junction** (43)**, drawn in cartoon form, with the strand cross-over point in the center indicated by the *arrow*.** The two B-DNA helices are inclined at an angle of ca 40°. PDB, Protein Data Bank.

DNA double helices can cross over each other and strand-exchange during recombination processes, as was first visualized by Holliday (41). Numerous Holliday junction crystal structures have been determined and are fully consistent with this genetic concept. They are typically formed from a single strand (for example a decanucleotide) and comprise four short helical stems linked at the center by a four-way branched junction (42, 43) (Fig. 5), having a right-handed twist. Each pair of B-form helices is approximately co-linear and even at the junction Watson–Crick base pairing is not disrupted. The cross-over of strands is achieved by a small number of backbone conformational angle changes in the nucleotides at the junctions. Holliday junctions can also be formed by telomeric sequences (44), which maintain the conservation of structure found in other Holliday junction structures and also have the conserved d(ACC) sequence at the cross-over.

## The surprising left-handed Z-DNA helix

The unequivocal assignment of right-handedness to A- and B-DNA double helices in the fibrous and crystalline states is in accord with the consistent right-handedness of DNA observed in a large body of protein complexes, from a wide range of functions (transcription factors, DNA repair complexes, helicases, topoisomerases, chromatin, etc). So, the reports in 1980 of the existence of a left-handed double helix with 12 nucleotides per complete helical turn, in crystals of alternating d(CG) sequences obtained under high-salt conditions (45, 46), came as a surprise and a shock. The arrangement, termed Z-DNA on account of the irregular features of the alternating CG backbone (Fig. 2*C*), was originally found in short tetra- and hexa-nucleotide duplexes and has now been observed in 162 structures of varying oligonucleotide length (PDB statistics as at 10 December 2020), as well as in a fiber diffraction analysis of an alternating d(CG) polynucleotide (47). The distinctive pattern of *syn*-G and *anti*-C nucleoside glycosidic angle conformations (1) is an essential requirement for Z-DNA, giving rise to the characteristic zig-zag arrangement for the phosphate groups. However, within an alternating d(CG) sequence, a degree of tolerance for the inclusion of alternating d(AT) base pairs is possible, albeit at the cost of some Z helix stability. Remarkably, many Z-DNA crystal structures are at exceptionally high resolution (48), even though flexible and even sometimes disordered phosphate groups are common.

Does Z-DNA have a biological function? The jury is still out on this, even 40 years after its discovery, although several well-established lines of evidence point to its involvement in numerous biological processes. Arguments have been proposed that it fulfils a function to maintain negative DNA winding, but perhaps more compelling have been the isolation and characterization of a number of Z-DNA binding proteins (49), notably several that are involved in immune responses (50), although the precise role of left-handed Z-DNA in these remains to be determined. Z-forming sequences are widely distributed in eukaryotic organisms, and they can induce genetic instability in both yeast and mammalian cells, which are resolved by the nucleotide excision repair pathway (51). Interestingly, this study used the PDB as the source of structural data to develop plausible molecular models for B-Z junction interactions with the ERCC1-XPF and MSH2-MSH3 repair protein assemblies: these were found to be supportive of the cleavage patterns and other experimental data in this study.

## DNA folding and higher-order arrangements

### Quadruplex DNAs

B-DNA duplex presence in the genome is not universal, especially when DNA is unwound during replication. However, at G-rich tracts, entirely different structures for DNA are possible. All the structures discussed above are (more or less) double-helical and require base-pairing à la Watson–Crick. The notion that DNA could be released from the constraints of the double helix and have a stable existence seemed heretical for many years, even though RNAs have long demonstrated their ability to form complex folded structures—t-RNAs, the ribosome, and ribozymes have many prominent features that are non-double helical, as well as A-RNA–type helical stems. It turns out that the history of non-duplex folded DNAs goes back over a century. The field is currently in a state of rapid advancement, encouraged by findings demonstrating that some of these structures can have biologically and therapeutically significant functions.

**Figure 6:**
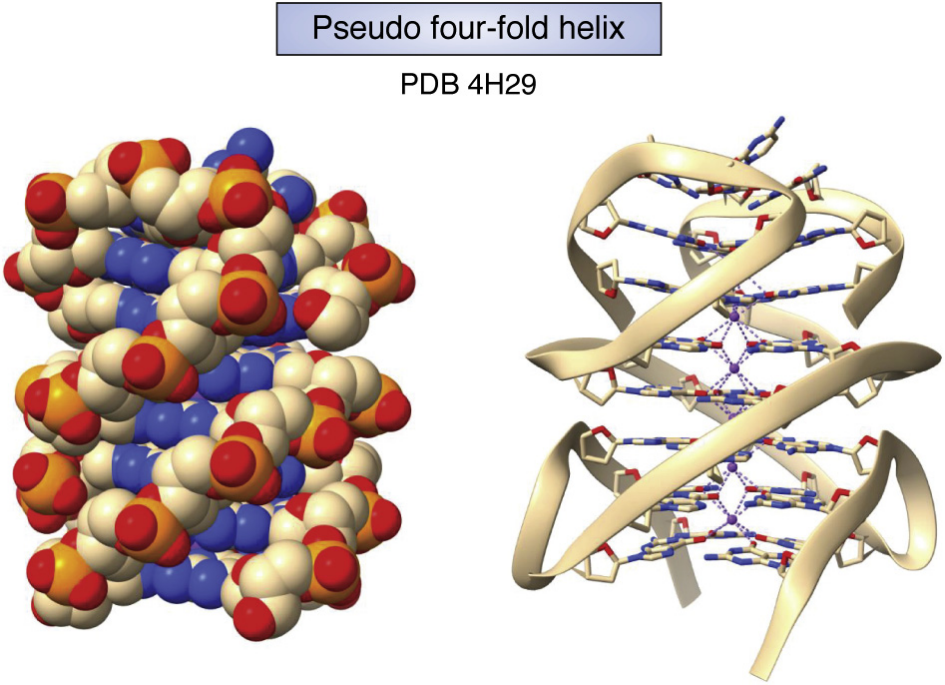
**van der Waals and cartoon depictions of a four-stranded right-handed quadruplex pseudo helix, as observed in the central core of seven stacked G-quartets in the crystal structure of the *BRAF* promoter quadruplex** (77)**.** All external looped-out adenine bases have been removed to enhance clarity. This bimolecular quadruplex is formed by two antiparallel strands, analogous to a duplex; however, these are highly G-rich and fold back to place the Gs in register and form the successive G-quartets and a four-stranded arrangement with four grooves. PDB, Protein Data Bank. Coordinated potassium ions are visible in the centre of the G-helix.

**Figure 7:**
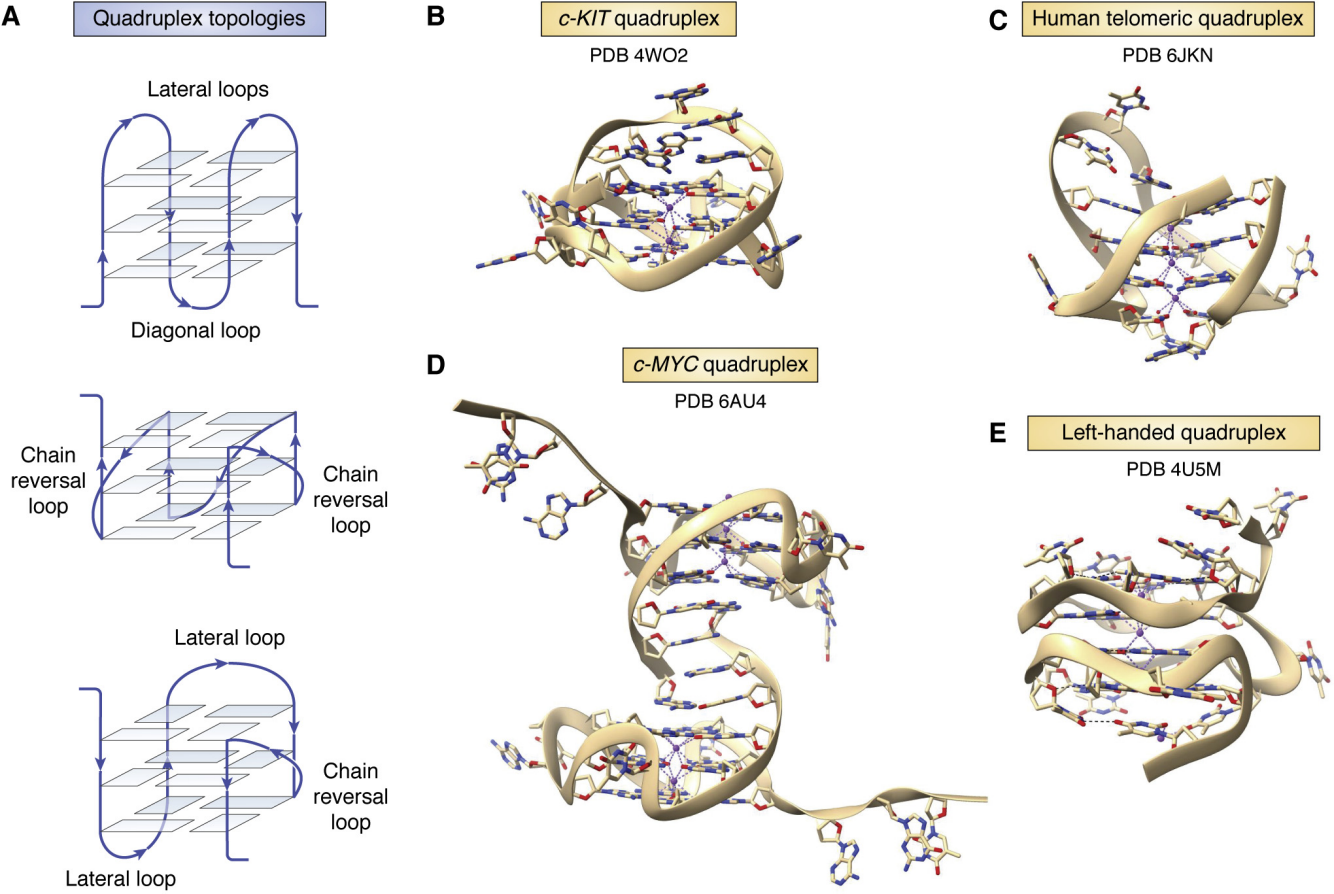
**Quadruplex folds.***A*, Schematic figures of three distinct quadruplex topologies, showing different loop types. The backbones in each case are colored *blue*, with strand directions shown by *arrows*. Human telomeric quadruplexes are polymorphic in solution and can adopt these and several other topologies (59–61). *B*, cartoon representation of the quadruplex formed from a promoter sequence in the c-*KIT* gene (76). Note the large A:G base-paired loop at the top of the structure. *C*, cartoon representation of the antiparallel chair arrangement formed by a human telomeric quadruplex (65), with three lateral loops. *D*, cartoon representation of the crystal structure of the c-*MYC* quadruplex (78). The two independent molecules in the crystallographic asymmetric unit are shown. Both have all-parallel topology, as in the human telomeric quadruplex (66), but with distinctive loops suitable for selective ligand binding. *E*, cartoon representation of a left-handed quadruplex (81). Note the narrow zig-zag groove parallel to the G-quartets. PDB, Protein Data Bank.

The ability of guanine bases to self-associate was first recognized in 1910 by the finding that G-rich guanosine monophosphate readily forms gels (52). The basis for this association was revealed, 60 years later, by fiber-diffraction studies, initially of gels formed from guanosine monophosphate (53). The diffraction patterns from these gels, which are analogous to the form in which fibers of A- and B-DNA can be formed, were consistent with a four-stranded helix comprising stacked G-quartets formed by four Hoogsteen hydrogen-bonded guanine bases (Fig. 1*C*). This novel structure was subsequently confirmed by fiber diffraction studies of poly r(G) (54, 55). Subsequent studies of telomeric DNA sequences, which comprise simple G-rich repeats such as d(TTAGGG) in human telomeres, has shown that these can fold into discrete stable structures, termed quadruplexes. Quadruplexes can be formed by intermolecular bimolecular association or by intramolecular folding, which also have (typically 2–4) stacked G-quartets, resulting in a stable four-stranded core (56, 57, 58) (Fig. 6). The short intervening sequences in a repeat [for example TTA in d(TTAGGG)] that are not directly involved in quartet formation can form extrahelical loops whose nature depends on the relative polarity of connecting strands as well as the length and nature of the loop sequence (6, 59, 60) (Fig. 7*A*). These factors, together with the possibilities of, for example, G-containing loops participating in G-quartet formation and quadruplexes being formed from multiple G-tract repeats, altogether imply many possible folds (61), of which only a fraction has been experimentally sampled to date. All quadruplexes have a requirement for an alkali metal ion, optimally potassium, to be coordinated to the inner-facing O6 atoms of G-quartets. Thus quadruplexes, and four-stranded G-helices (sometimes termed G-wires), all have a central ion channel (Fig. 6), ironically analogous to this feature in some of the early incorrect predouble helix models of DNA itself.

Human telomeric quadruplexes, formed from repeats of the sequence d(TTAGGG), have been much studied by crystallography and NMR, showing that two to four repeats can fold into a variety of topologies, depending on factors such as flanking sequence, nature of the cation, and concentration. Those structures observed in more dilute solution can have antiparallel, chair, basket, or hybrid topologies, with various combinations of lateral, diagonal, or chain reversal d(TTA) loops (see for example, refs. (62, 63, 64, 65) and Fig. 7*C*), whereas at higher concentrations and in the crystal, the all-parallel more compact form has only chain-reversal (propeller) d(TTA) loops (66). There is continuing controversy as to which form of telomeric quadruplex has greater biological relevance to telomeres *in situ*, with some evidence that molecular crowding, as found in the cell nucleus, would favor the parallel form (see for example, ref. (67)).

Bioinformatics searches have revealed that potential quadruplex-forming sequences occur widely but nonrandomly in the human and other genomes (68, 69), with over-representation in the promoter regions of many cancer and proliferative-associated genes (70, 71, 72). These searches have used a general formalism for “simple” quadruplex sequences is G_m_X_n_G_m_X_o_G_m_X_p_G_m_, where m is the number of G residues in each short G-tract, and X_n,o,p_ represents general-sequence loops. Numerous roles for quadruplexes have been identified, especially in transcription, translation, and replication, and consequently, many quadruplexes are potential therapeutic targets, especially for genes involved in human cancers (72, 73, 74, 75).

Quadruplex structures from human promoter sequences have been extensively characterized by crystallography and NMR methods (see for example, Table 1 and Fig. 7, *B* and *D*). Many of these structures are based on a parallel strand topology, as initially observed in the crystal structure of the human telomeric quadruplex (66), especially when at least one loop comprises a single nucleotide. The number of guanines in each short G-tract can vary, with those guanines not incorporated in the G-quartets, sometimes being part of the loops, resulting in complex folds such as that from the c-*KIT* gene (76) (Fig. 7*D*). The crystal structures of the *BRAF* (77) (Fig. 6), c-*MYC* (78) (Fig. 7*D*), and *KRAS* (79) promoter quadruplexes (Table 1) are examples of quadruplexes with the potential to be therapeutic targets in human cancers. Quadruplexes are also prevalent in the genomes of other organisms, and there is especial current interest in their potential as antiviral targets (80).

The precise number of quadruplexes encoded in the human genome is still a matter of lively debate, and it is likely that many have only a transient existence, especially if they are constrained within nucleosomes; however, an estimate of ca 10,000 for quadruplexes in active chromatin in cells (72) is probably realistic. Although there are currently (as of 15th January 2021) 520 quadruplex entries in the PDB, only a small number represents “human genomic quadruplexes”, and several recent structure determinations have emphasized the point that the complexity of quadruplex folding is very incompletely understood. Notable examples of a hitherto unexpected topology are the NMR and crystal structures of left-handed quadruplexes (Z-G4s) (81, 82), where small changes in the sequence of a close analog of the well-studied (83) anticancer DNA aptamer AS1411 (which itself forms a right-handed parallel-stranded quadruplex (84)) result in a dramatic reversal of backbone and helix polarity. This left-handed quadruplex has a single highly extended and narrow groove that is almost parallel to the G-quartet plane and which winds around ca 90% of the quadruplex helical core (Fig. 7*E*). The uneven backbone is reminiscent of that of Z-DNA. A small number of occurrences of the Z-G4 sequence have been mapped in the human genome (81), but it would be unsurprising if other sequence types capable of forming left-handed quadruplexes are to be found in the future.

### DNAs as enzymes, large, and small

The well-established ability of RNAs to fold into complex arrangements (ribozymes) possessing catalytic activities against a range of substrates prompts the question of whether quadruplex (and other) folded DNAs can perform analogous functions. The answer is yes (85), perhaps more often and with greater variety than was initially envisaged. It has been shown (86) for example, using *in vitro* selection, that certain quadruplexes complexed with a hemin group can possess sufficient peroxidase enzymatic activity to oxidize a range of organic substrates such as indoles. Quadruplex-hemin complexes can also show NADH oxidase and NADH peroxidase activity (87). More recently (85), the concept has been greatly extended by several demonstrations of asymmetric catalysis using a quadruplex together with an appropriate metal complex to achieve Diels-Alder, Michael addition, and Friedel–Crafts alkylations. There is little structural information to further develop progress in this area, although several studies have indicated the importance of quadruplex topology (85).

**Figure 8:**
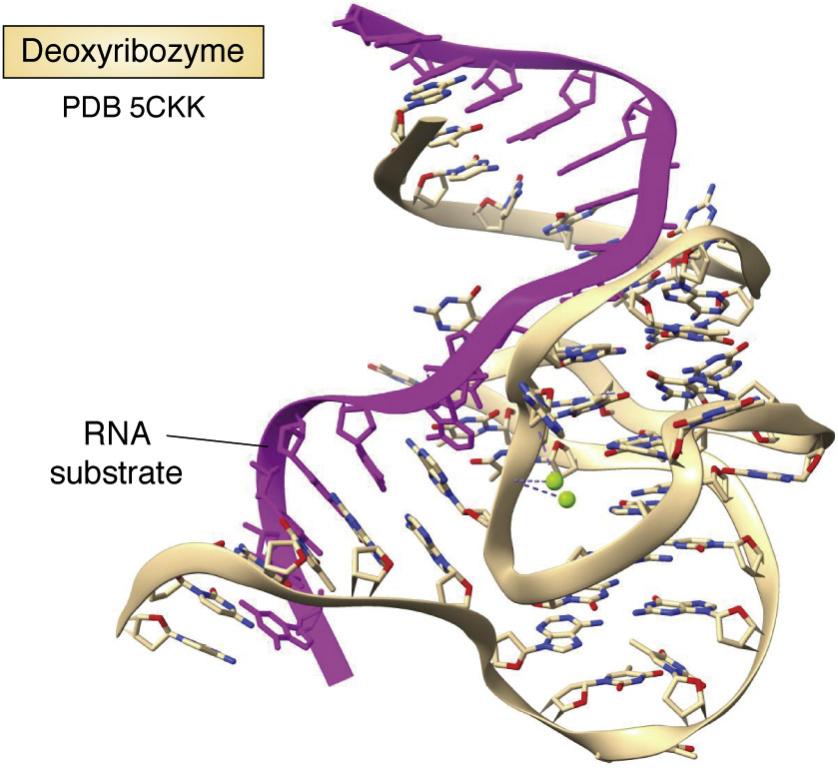
**Ribbon cartoon representation of the crystal structure of a deoxyribozyme** (91)**.** Its RNA substrate sequence is colored *purple*. PDB, Protein Data Bank.

Deoxyribozymes (DNAzymes), the analogues of ribozymes, were discovered by *in vitro* selection and have analogous, complex tertiary structures (see for example, Fig. 8). The first one identified (88) is able to bind to and catalytically cleave RNA sequences, and the large number (over 1500 (89, 90)) that have been subsequently found display a range of other catalytic activities as well, including not only the expected RNA ligation and DNA hydrolysis but can also catalyze a range of organic chemistry reactions—Diels-Alder, phosphorylation, and glycolysis to name just a few. Metals can be involved in the various catalytic mechanisms but are not universally required. Crystal structures of several DNAzymes are available, with a recent one (91) having RNA-ligating activity and revealing the structural features relevant to catalysis (Table 1 and Fig. 8), including the core requirement of 31 nucleotides. This 44-nucleotide DNAzyme is complexed to a 15-mer RNA, whose ends are hybridized to the DNA, forming two heteroduplexes with the complex hydrogen-bonding and base-stacking arrangement between them, forming the active site. DNAzymes do not appear to have many natural biological functions, although their potential for use as catalysts is considerable.

## The importance of the PDB and NDB for studies on DNA structure, flexibility and function

The determination of a single DNA crystal structure is normally only the starting point in a journey of analysis and comparison, as indicated by the examples given in this review. This requires full access to coordinate data for previously determined structures and software tools for effectively examining and comparing them. Soon after the appearance of the first nucleic acid fragment crystal structures, it became apparent that the lack of freely available coordinate data for some of these structures was becoming an issue for many, not least those in the biological community as the importance of structure became increasingly widely appreciated. Some, though not all, of the early oligonucleotide crystal structures had been deposited with the Cambridge Crystallographic Centre, which (still) is the world-wide repository for small-molecule crystal structures, but which at that time ran a subscription-based service. The infant PDB, with its commitment at the outset to full and open access, soon became the depository of choice for all oligonucleotide structures. Its expertise in these structures has facilitated robust validation tools to be developed that have enabled (i) the PDB structure depositors and users to check on the quality and detailed data features for deposited crystal or NMR structures and (ii) crucially all those interested in a structure to be confident of its quality and even very occasionally, its correctness. An example shows the power of the PDB validation tools for two reported crystal structures of the *Oxytricha nova* bimolecular quadruplex sequence d(G_4_T_4_G_4_). Crystal structure 1D59, reported in 1992 (92) at 2.3 Å resolution, shows an antiparallel hairpin dimer quadruplex, whereas the subsequent structure 1JPQ for the identical sequence, reported in 2002 (93) at 1.6 Å resolution, shows a distinct antiparallel diagonal loop arrangement. The PDB validation reports on the two structures reveal that 1D59 has 86 close contacts in the crystal structure, compared with three in 1JPQ. The discrepancy between the two structures has been ascribed to errors in the phosphodiester chain tracing for 1D59, leading to a high number of close contacts. The structure assignment in 1JPQ is in accord with independent NMR studies of this sequence in solution (94) and in a protein complex (95), further evidence supporting the contention that the 1JPQ structure is the correct one.

The degree of “correctness” of a crystal or NMR structure, *i.e.,* its accuracy and precision, is of paramount importance to the large number of derived studies on DNA and other nucleic acid structures, *e.g.,* in theoretical and computational studies, in nanoscience, in drug design and discovery, and of course in molecular and cellular biology. The widespread use of computational methods to model structures and intermediates, *e.g.,* in DNA folding, using chiefly molecular dynamics simulations, has highlighted the need to constantly improve the available force fields so that computational results can accurately reproduce good-quality experimental structural data (96, 97, 98, 99), especially in terms of backbone/sugar conformations and base/base pair morphology. Force field and geometric parameterizations for bases, sugars, and phosphodiester backbones are also of critical importance for reliable crystallographic and NMR structure refinements, and an early database compilation (100) of consensus experimental DNA and RNA geometry is still of value.

For those who want to examine structures in more detail, the NDB, established in 1992, directly provides further information compared with the PDB on a given structure, with data on (i) backbone torsional angles, (ii) sugar conformations, (iii) base morphological parameters, and (iv) base pair hydrogen bonding. All this information provides a goldmine for comparative studies on these factors, enabling trends in structure (and in other features such as DNA hydration) to be statistically assessed for large groups of structures (see, for example, refs. (101, 102, 103, 104)). Such data-mining studies have revealed for example that in duplex DNA sequences, the d(TA) step is the most flexible in terms of base step morphology parameters such as roll and tilt and thus can contribute to DNA helix bending (21, 26, 27, 28, 29, 30, 31).

**Figure 9:**
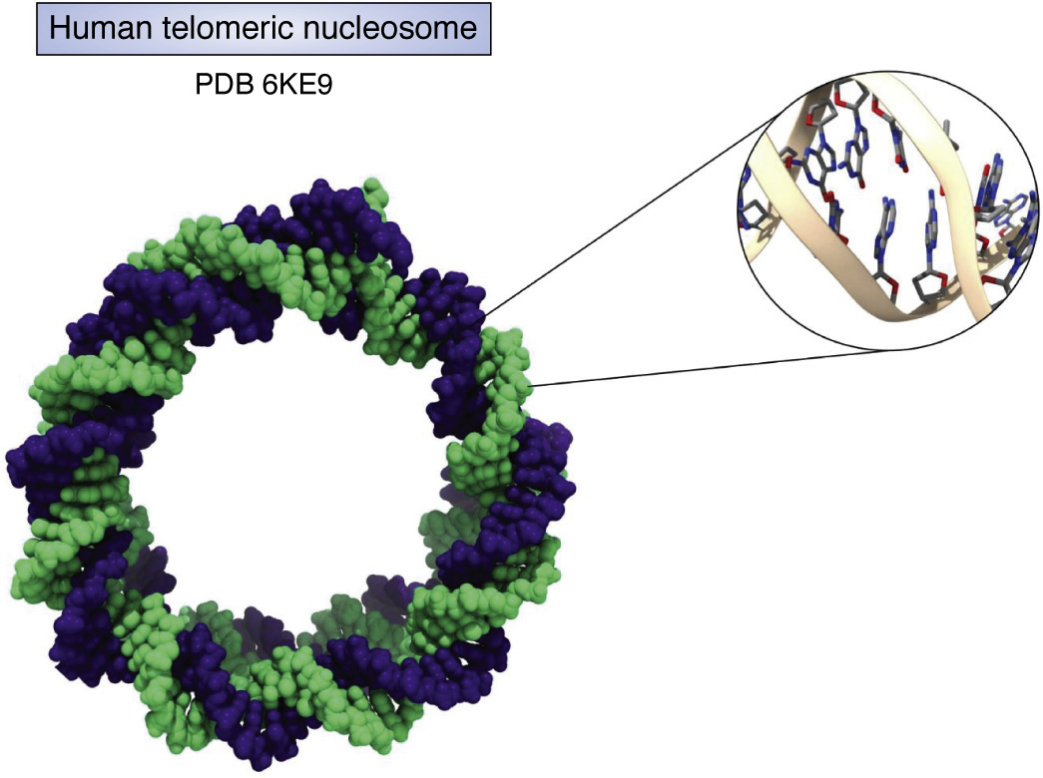
**The structure of the DNA component of a human telomeric nucleosome** (105)**, shown in space-filling representation with the two strands in separate colors, together with a magnified view of a typical region of DNA curvature, showing changes from ideality in base pair morphology such as roll, tilt, and twist**.

This review commenced with a discussion of the classic B-DNA double helix structure; we conclude with two examples of studies on B-DNA deformations from ideality and how data from the PDB has complemented findings from other experimental and theoretical approaches. The B-DNA helix bending in the crystal structure (105) of a nucleosome comprising human telomeric DNA (with 23 (TTAGGG) repeats), in which the flexible d(TA) step, as well as d(TT) ones, plays an important role in facilitating the bending necessary for wrapping around the histone core, as shown in Figure 9. DNA bending is related to minor groove width, which has been extensively studied in solution (106). The use of nucleotide-resolution hydroxyl radical foot-printing is a powerful gene (and genome) mapping probe, especially when allied to considerations of experimental structural data on parameters such as groove width. This technique has shown that in seven out of 11 protein–DNA complexes, the minor groove width is in accord with that observed in the native DNA sequences (107) in solution and in the crystal. In other words, groove width is an intrinsic property of many DNA sequences, and one can have confidence that crystallographic studies on native sequences can reflect this reality. Of course, many DNA-binding proteins distort DNA structure, but these distortions can reflect the tendencies observed in the native DNA structures, as shown by simulation studies on tetranucleotide sequences embedded within the DNA structures contained in the PDB (27). These studies also highlight that only a small number of the total tetranucleotide sequences (5 out of 136) are represented experimentally in native DNA sequences in the PDB; even including protein-DNA complexes, only added 19 sequences to this list, as at December 2019. Clearly much more experimental structural data are needed before we have a comprehensive understanding of DNA flexibility.

**Figure 10:**
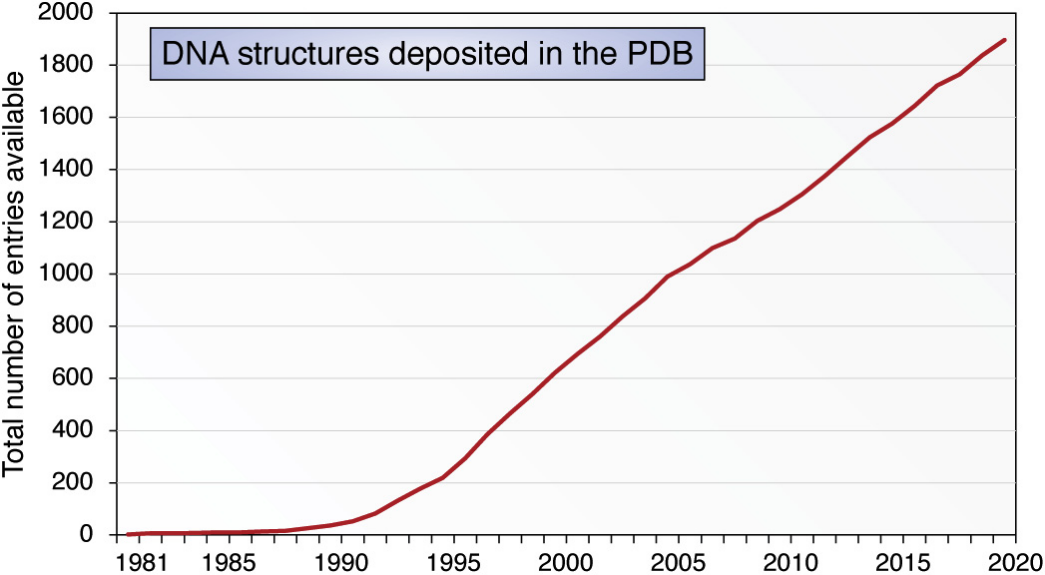
**Year-on-year increases in the number of DNA structures deposited in the PDB, in the period 1981 to 2020.** I am grateful to Dr Cathy Lawson, the current Director of the NDB, for this information. NDB, Nucleic Acid Database; PDB, Protein Data Bank.

The PDB is for most of us the source of information where one can rapidly download structure(s) of interest and/or simply examine them visually, using the built-in visualization tools of the PDB. There are ca 2000 experimental DNA structures currently in the PDB (Fig. 10) with the numbers continuing to increase. The variety of DNA structures in the PDB is reflected in the realization that these can have profound biological roles in addition to that of DNA as an information depository, and whatever they are, one can be confident that the future will disclose further novel structures and attendant biology.

## Conflict of interest

The author declares that he has no conflicts of interest with the contents of this manuscript.
